# High Nitrogen Availability Limits Photosynthesis and Compromises Carbohydrate Allocation to Storage in Roots of *Manihot esculenta* Crantz

**DOI:** 10.3389/fpls.2019.01041

**Published:** 2019-09-11

**Authors:** John Okoth Omondi, Naftali Lazarovitch, Shimon Rachmilevitch, Uri Yermiyahu, Or Sperling

**Affiliations:** ^1^French Associates Institute for Agriculture and Biotechnology of Drylands, Ben-Gurion University of the Negev, Midreshet Ben-Gurion, Israel; ^2^Gilat Research Center, Agricultural Research Organization, Gilat, Israel

**Keywords:** root-crops, nitrogen, fertigation, carbohydrates, physiological indicators

## Abstract

Cassava (*M. esculenta* Crantz), feeding countless people and attracting markets worldwide, is a model for traditional crops that need physiology-based fertigation (fertilization through irrigation) standards in intensive cultivation. Hence, we studied the effects of 10 to 200 mg L^-1^ nitrogen (N) fertigation on growth and yields of cassava and targeted alterations in their photosynthetic, transpiration, and carbohydrate management. We found that increasing irrigation N from 10 to 70 mg L^-1^ increased cassava’s photosynthesis and transpiration but supported only the canopy’s growth. At 100 mg N L^-1^ cassava reached a threshold of sugar in leaves (∼47 mg g^-1^), began to accumulate starch and supported higher yields. Yet, at 200 mg N L^-1^, the canopy became too demanding and plants had to restrain transpiration, reduce photosynthesis, decrease carbohydrates, and finally lower yields. We concluded that the phases of cassava response to nitrogen are: 1) growth that does not support yields at low N, 2) productive N application, and 3) excessive use of N. Yet traditional leaf mineral analyses fail to exhibit these responses, and therefore we propose a simple and inexpensive carbohydrate measurement to guide a precise use of N.

## Introduction

With increased pricing of fertilizers and rising environmental concerns, the effectivity of nutrient application has to be studied thoroughly for every new crop, condition, and farming practice. It is especially critical in arid and semi-arid environments, where the shift to intensive fertilization through irrigation (i.e., fertigation) alters the nutrient uptake and use and requires specific considerations (He et al., 2015). Of all the macro-elements, modern farming relies heavily on nitrogen (N), which, despite becoming increasingly costly, cannot be substituted by farmers. Nitrogen-deficient plants halt leaf elongation (Marschner and Marschner, 2012), inhibit photosynthesis (Gregoriou et al., 2007), reduce chloroplast size (Li et al., 2013), and minimize overall growth. Yet, if N levels increase and it is no longer a limiting factor in physiological processes, its effectivity must be reassessed. In fact, the majority of applied N in crop systems often does not transform to increased yield (Lassaletta et al., 2014) due to numerous parameters that affect its effectivity, e.g. crop species and variety, the N form (nitrate or ammonium), soil type, water availability, and application method (El-Sharkawy et al., 1998; Ospina et al., 2014). Thus, N should be studied within the conditions and limitations of the entire farming environment to ensure its application supports yield and profits.

Cassava (*Manihot esculenta* Crantz) cultivation is expanding, and its intensive cultivation needs to be studied thoroughly. This South American native (Nassar, 2007) is the third largest source of carbohydrates in the tropics and the sixth most important crop worldwide (Lebot, 2008). It is not only a food staple to over 500 million people, but an important constituent in commercial animal feed, paper or textile fibers, and starch for pharmaceutical industries (Balagopalan, 2002; Tonukari et al., 2015). Due to water scarcity, cassava farms are shifting to drip irrigation, and production could increase further by better soluble nutrients application (Susan John et al., 2007; Fermont et al., 2009; Byju et al., 2012). So far, we know that N fertilization increases N concentration in the leaf blade (Nguyen et al., 2002), leaf area index (Sangakkara and Wijesinghe, 2014), photosynthetic rate (Cruz et al., 2003b), plant height, number of leaves, stem diameter, and the number of roots (Uwah et al., 2013) of cassava. However, the effect on root yield is arguable, as earlier studies suggested that N application induces cassava shoot growth at the expense of storage root formation (Cenpukdee and Fukai, 1991) and starch content (Cruz et al., 2003a). More recently, Nguyen et al. (2002) observed a yield decline at 160 kg N ha^-1^ for the first year of application, while Kaweewong et al. (2013) reported optimal root yields at N applications as high as 250 kg N ha^-1^. Conversely, Uwah et al. (2013) showed no yield response to an increase from 80 to 120 kg N ha^-1^ whatsoever. These studies demonstrate that cassava responds to N fertilization and that yields can improve but emphasize that physiological bases for efficient N use are still obscure.

Matching N application to crops’ requirements calls for studying its specific physiological effects. As N constitutes a part of the carbon sinks (e.g. amino acids and proteins), it has a positive effect on photosynthesis and transpiration (Leuning et al., 1995; Bar-Tal et al., 2001). Nitrogen is critical initially at the leaf level, where it improves radiation-use efficiency and promotes photosynthetic productivity (Sinclair and Horie, 1989). Yet high levels of photosynthates, without adequate transport, can signal that the capacity for non-structural carbohydrates (NSC) has reached its maximum, and down-regulate photosynthesis (Goldschmidt and Huber, 1992). Moreover, N tends to promote vegetative growth (Kang et al., 2004), increase a plant’s root-to-shoot ratio (Grechi et al., 2007), and might exhaust the roots’ water delivery capacity. Indeed, it is low N that promotes root growth (critical for root-crops as cassava) and supports high transpiration demands (Marschner and Marschner, 2012). In a water-limiting environment, high N levels could restrain water losses and the corresponding CO_2_ sequestration, and force plants to utilize their residual photosynthates in the canopy at the expense of roots and reproduction. This implies that besides growth, water, and nutrient uptake, NSC levels could indicate whether the N application is effective.

Consequently, we studied N application in fertigated cassava and the physiological implications of high N availability. We postulated that if N supports shoots over root development then root-crops (e.g. Cassava) are prone to over-fertilization. Our objectives were therefore to 1) define physiological responses in N fertigation levels ranging from 10 to 200 mg L^-1^ and 2) determine a physiological indicator to the effectivity of N application on cassava yields. We used inert perlite, which does not buffer the minerals, and studied the presence and form of N in leaves. We also targeted the processes that drive the starch accumulation in roots of cassava, i.e., photosynthesis and carbohydrate management to form new applied indexes.

## Materials and Methods

### Setup

We experimented with 30 potted cassava plants (*Manihot esculenta* Crantz cv. Israeli) in a greenhouse at Gilat Research Center, Israel (31° 20´ N and 34° 39´ E) during the summer of 2014 (Exp. 1). The average humidity in the greenhouse was 80%, radiation 136 W m^-2^, and temperature 23°C. Plants were trimmed in mid-season (100 days after growth) and therefore we could not report on total biomass in Exp. 1 (we did include the rest of the growth parameters from Exp. 1 in the final analysis). Hence, we repeated the experiment in the summer of 2015 (Exp. 2), included final canopy biomass, and reinforced our findings. In both experiments, cassava cuttings (20 cm long) were planted with 6 nodes in 2-L pots (a single cutting per pot) filled with 6 mm perlite granules with high porosity, high hydraulic conductivity, and minimum interaction with nutrients (Erel et al., 2015). For the first 28 days, plants were hand-irrigated every 48 h with 500 ml nutrient solution (100 mg N L^-1^, 10 mg P L^-1^, 100 mg K L^-1^, 60 mg Ca L^-1^, 30 mg Mg L^-1^, and microelements). Then, plants (now having ∼5 leaves) were transplanted in 60-L pots filled with perlite, and the N concentration in the fertigation changed to either 10, 40, 70, 100, 150, or 200 mg N L^-1^ (0.7, 2.9, 5, 7.1, 10.7, 14.3 mM N, respectively). There were 5 plants per treatment in a complete randomized design, the concentrations of microelements in the irrigation did not change, and the irrigation quantity was fixed periodically (by collecting and weighing drainage for 24 h) to ensure a 0.3 leaching fraction.

### N Solution Preparation

Stock solutions for the experiment were made of dissolved NH_4_NO_3_, NH_4_H_2_PO_4_, KH_2_PO_4_, K_2_SO_4_, KNO_3_, NaNO_3_, and MgCl_2_ in tap water [electrical conductivity (EC) of 0.3-0.4 dS m^-1^]. Then, stock solutions were diluted by 200 times in 1,200-L tanks to final fertigation solutions. These final solutions were also supplemented with Mg, Ca and microelements (60 mg Ca L^-1^, 30 mg Mg L^-1^, 8,0 mg Fe L^-1^, 4.0 mg Mn L^-1^, 2.0 mg Zn L^-1^, 0.4 mg B L^-1^, 0.3 mg Cu L^-1^, and 0.22 mg Mo L^-1^) and the proportions of NO_3_^-^ to NH_4_^+^ were always fixed at 9:1. The stock and final solutions were regularly sampled and analyzed for NO_3_^-^, NH_4_^+^, P, and K to verify if they were within 5% of the anticipated levels, while the pH and the EC in the final solution were checked weekly and ranged at 6.9 ± 0.3 and 0.95 ± 0.1 dS m^-1^, respectively.

### Measurements and Harvest

The youngest fully expanded leaves (diagnostic leaf) were sampled at 29, 51, and 88 days of treatment (DOT) and separated to blade and petiole for mineral analysis. Photosynthesis (A_n_, LI-6400, Li-Cor Inc., Lincoln, NE, USA) and stomatal conductance (g_s_, LI-6400) of matured, sun-exposed leaves were measured after 91 days of growth in three plants per treatment between 10:00 and 13:00. The reference CO_2_ in the LI-6400 was set to 400 ppm, light to 1,000 µmol m^-2^ s^-1^, and temperature and humidity levels were ambient (∼30°C and 40% RH in the greenhouse). All the plants were harvested after 190 days of growth, divided into tuberous roots, stem, and leaves. The total fresh weight was recorded and all the samples were oven-dried at 70°C for 48 h, and they were then re-weighed to ascertain their water content and dry mass (DM). Finally, leaves of 18 plants (3 replicates per treatment) were stored for subsequent lab carbohydrate analysis.

### Laboratory Analysis

Dried leaf samples were ground in a stainless-steel coffee mill to 0.5 mm particle size and measured for total N in the leaf blade by digestion in sulfuric acid and peroxide (Snell and Snell, 1949). The NO_3_^-^ portion was also determined in the leaf petiole (Yermiyahu et al., 2017) by weighing 100 mg of dry powder, mixing it in 10 ml DI for 30 min, filtering it through paper (Whatman 42), and detecting the concentration by an automated photometric analyzer (Thermo Scientific Gallery Plus).

Non-structural carbohydrates (NSC) in leaves were extracted by an updated version of Leyva et al. (2008) from finely ground (MiniBeadbeater-96, Glen Mills Inc., NJ) tissues. A 25 mg portion of the dry leaf sample was mixed with 550 µl Na acetate buffer (pH 5.8) and shaken for 15 min at 72°C in 1.5 ml tubes. The insoluble tissue was precipitated in a centrifuge (17,000 g) and 50 µl of the supernatant was collected for soluble carbohydrate (SC) analysis. The samples were then hydrolyzed for 20 min at 100°C, cooled to room temperature, and incubated with 100 µl amyloglucosidase and 100 µl α-amylase for 4 h at 37°C (while shaking) to digest starch. The mixture was reprecipitated in the centrifuge (17,000 g) and another 50 µl of supernatant was collected for the starch analysis.

To quantify carbohydrates, the 50 µl supernatants (for both SC and starch) were diluted in 1 ml of DI. Then 50 µl of the solutions were mixed in a 96-well micro-plate with 150 µl anthrone and 98% sulfuric acid (0.1% w/v). The plate was heated to 100°C for 10 min in a water bath, cooled to room temperature for another 10 min, and the absorbance at 620 nm was measured in a plate reader (Multiskan GO, Thermo Scientific, Finland). The readings were compared to a linear regression of glucose standards (0.01, 0.03, 0.1, and 0.3 g L^-1^), and the equivalent SC concentration was computed. The original supernatant collection stood for SC concentrations in the samples, while the delta between the readings before and after the enzymatic reaction represented the starch levels.

### Statistics

The variance of the results was tested by a one-way ANOVA, and the means were compared by LSD (95% confidence) using the SAS software (SAS, 2002). Relations between transpiration and photosynthetic performance, and between dry root yield and leaf carbohydrates (soluble sugar and starch), were assessed by linear regression.

## Results

### Growth, Yield, and N Diagnosis

**Figure 1:**
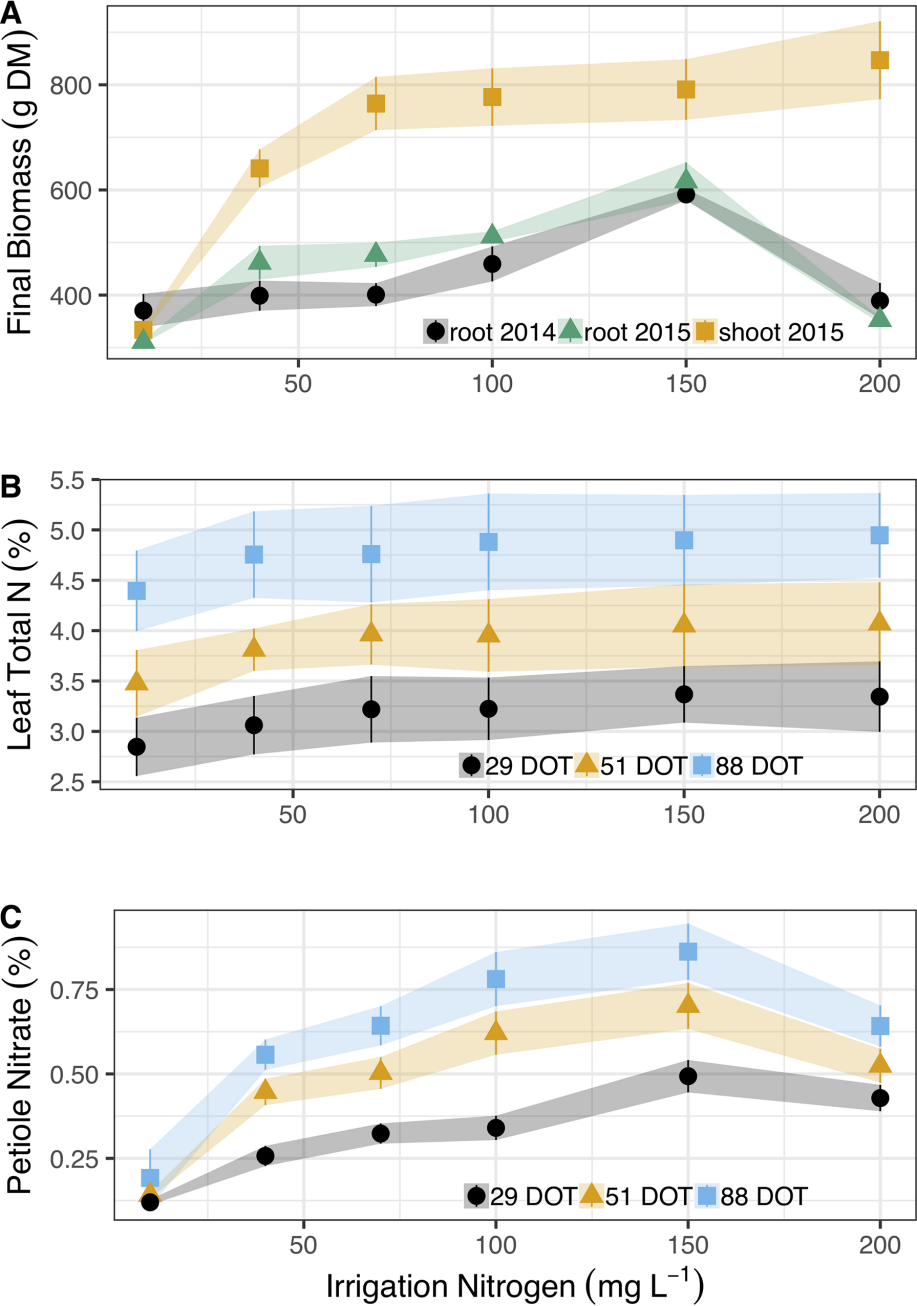
Irrigation N concentrations’ (10, 40, 70, 100, 150, and 200 mg L^-1^) effect on **(A)** Dry shoot mass accumulation (g) in 2015 (yellow squares) and dry root yields in 2014 (black circles) and 2015 (green triangles). **(B)** Total N concentrations (%) in the leaf blade after 29 (black circles), 51 (yellow triangles), and 88 (blues squares) days of treatment (DOT). **(C)** Corresponding nitrate concentrations (%) in the leaf petioles of cassava. Shapes represent means (five biological replicates) whereas vertical bars and ribbons mark SE.

Cassava plants doubled their shoot mass (from 330 to 760 g DM plant^-1^) as N irrigation levels increased from 10 to 70 mg L^-1^ (**Figure 1A**). However, shoots retained similar mass (∼780 g plant^-1^) at 70, 100, and 150 mg N L^-1^. At 200 mg N L^-1^ in irrigation, cassava plants induced shoot mass, to 850 g plant^-1^ (0.1 > P > 0.05). Nitrogen did not have such a positive effect on cassava root between 10 and 100 g N L^-1^ in irrigation, as they maintained an average mass of 407 g in Exp. 1 and 440 g in Exp. 2 (despite an increase between 10 and 40 mg N L^-1^). Roots induced mass at 150 g N L^-1^ and reached ∼600 g plant^-1^ in both years. Yet most importantly, 200 mg N L^-1^ in irrigation severely inhibited cassava root development and reduced mass significantly to 389 g plant^-1^ and 352 g plant^-1^ at Exp. 1 and Exp. 2, respectively.

Diagnostic leaves of cassava did not indicate such dramatic effects of N on cassava roots or canopy development (**Figure 1B**, detailed statistics at **Table 1**). In fact, the only differences appeared between the sampling dates (**Table 1**) as N concentrations in diagnostic leaves increased from 3.2% to 3.9%, and 4.8% (±0.2%) between the 29^th^, 51^st^, and 88^th^ days of treatments. It is important to note that we always sampled young fully expanded leaves and these differences are not the net accumulation of N in individual leaves. Nitrate in the petiole, i.e., unfixed N deposited from sap, did respond to irrigation N and its concentrations gradually increased from 0.19% to 0.86% between 10 and 150 g L^-1^ at the 88^th^ day of treatment, and then decreased to 0.64% at 200 mg N L^-1^ (**Figure 1C**). At the 29^th^ and 51^st^ days of treatment nitrate concentrations likewise increased from ∼0.13% to 0.5% and 0.7% (29^th^ and 51^st^, respectively), and decreased to ∼0.47% with the 200 mg N L^-1^ treatment.

**Table 1:** One-way ANOVA for Total N (%) and Nitrate (NO_3_^-^, %) in leaves of cassava after 29, 51, and 88 days of irrigation (days of treatment – DOT) with 10, 40, 70, 100, 150, and 200 mg N L^-1^.

Fertigation (mg N L^-1^)	DOT 29		DOT 51		DOT 88	
	Total N (%)	NO_3_^-^ (%)	Total N (%)	NO_3_^-^ (%)	Total N (%)	NO_3_^-^ (%)
10	2.85 A	0.12 D	3.48 A	0.14 C	4.4 A	0.192 C
40	3.06 A	0.26 C	3.81 A	0.45 B	4.76 A	0.56 B
70	3.22 A	0.32 B	3.96 A	0.50 AB	4.76 A	0.64 AB
100	3.22 A	0.34 B	3.95 A	0.62 A	4.88 A	0.78 A
150	3.37 A	0.49 A	4.05 A	0.7 A	4.9 A	0.86 A
200	3.34 A	0.43 A	4.07 A	0.52 AB	4.95 A	0.64 AB

Capital letters denote comparison of means by Tukey-LSD (5 biological replicates, P < 0.05) in total N or NO_3_^-^ levels at DOT 29, 51, or 88 independently.

### Photosynthetic Attributes

**Figure 2:**
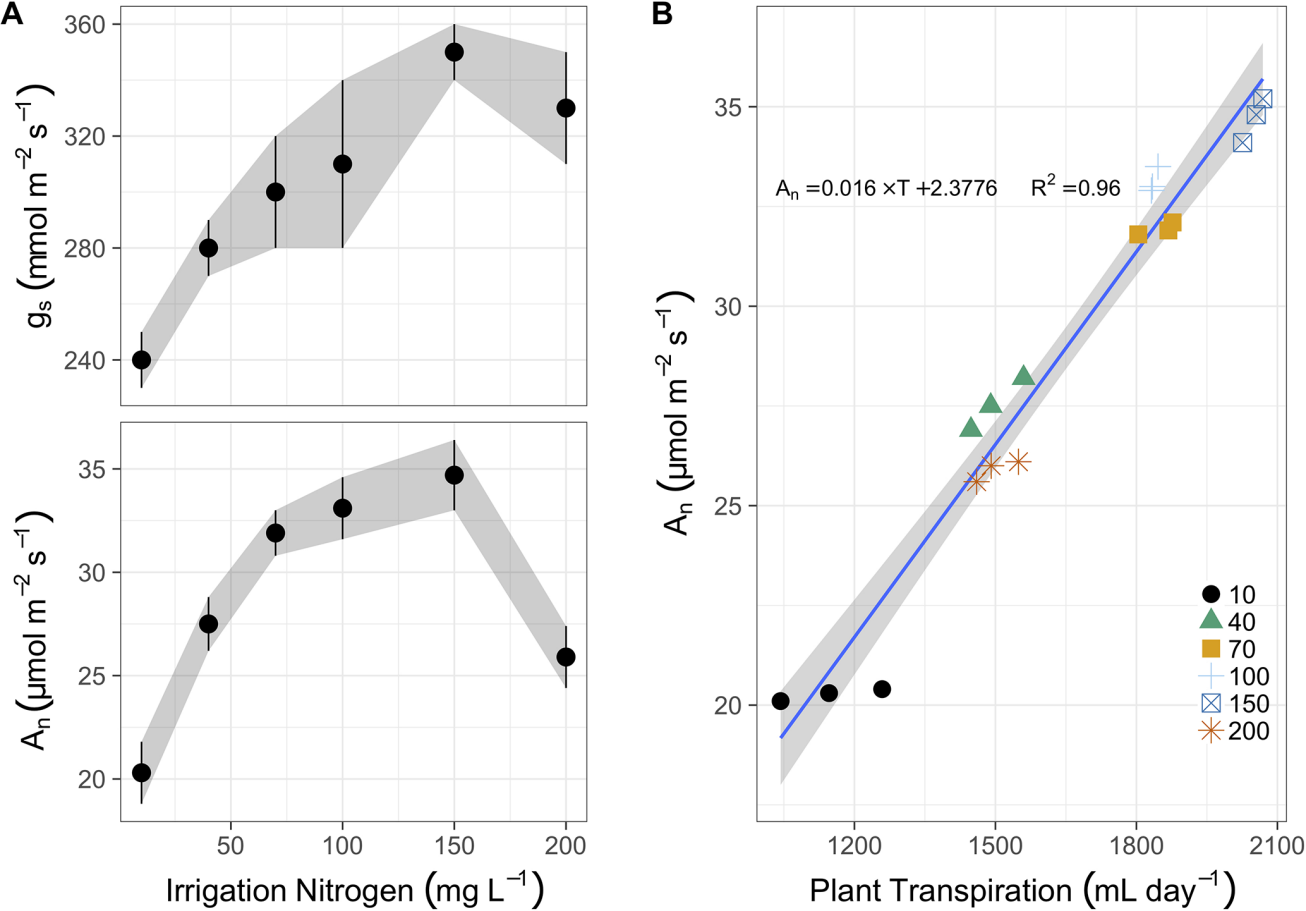
**(A)** The response of stomatal conductance (g_s_, top panel, mmol m^-2^ s^-1^) and photosynthesis rate (A_n_, bottom panel, µmol m^-2^ s^-1^) after 100 days of N fertigation at concentrations from 10 to 200 mg L^-1^ (means of 3 biological replicates ± SE). **(B)** The correlation between photosynthesis and cumulative daily transpiration (ml day^-1^) for N concentrations from 10 to 200 mg L^-1^ (represented by various shapes and colors in legend), the linear regression (blue line and text for equation), and the 95% confidence intervals (grey ribbon).

Midday photosynthesis increased from 20 to 32 µmol m^-2^ s^-1^ between 10 and 70 mg N L^-1^ in irrigation (**Figure 2A**). Then it increased gradually (though not significantly different) to 35 µmol m^-2^ s^-1^ at 150 mg N L^-1^, and finally dropped sharply to 26 µmol m^-2^ s^-1^ at 200 mg N L^-1^. Stomatal conductance also increased due to N application, almost linearly between 10 and 150 mg L^-1^ (240 to 350 mmol m^-2^ s^-1^), followed by a minor drop to 330 mmol m^-2^ s^-1^ at 200 mg N L^-1^. The photosynthetic rate was best correlated to the total daily transpiration (**Figure 2B**). It increased linearly from ∼20 µmol m^-2^ s^-1^ to ∼35 µmol m^-2^ s^-1^, as transpiration augmented from 1,150 g day^-1^ to 2,050 g day^-1^ (over 2 L a day per plant). Yet the N treatments did not dictate this correlation, as plants irrigated with 200 mg N L^-1^ transpired like plants irrigated with 40 mg L^-1^, and even photosynthesized slightly less (26 µmol m^-2^ s^-1^ vs. 27.5 µmol m^-2^ s^-1^), i.e., well below the 70, 100, and 150 mg N L^-1^ treated plants.

### Non-Structural Carbohydrates

**Figure 3:**
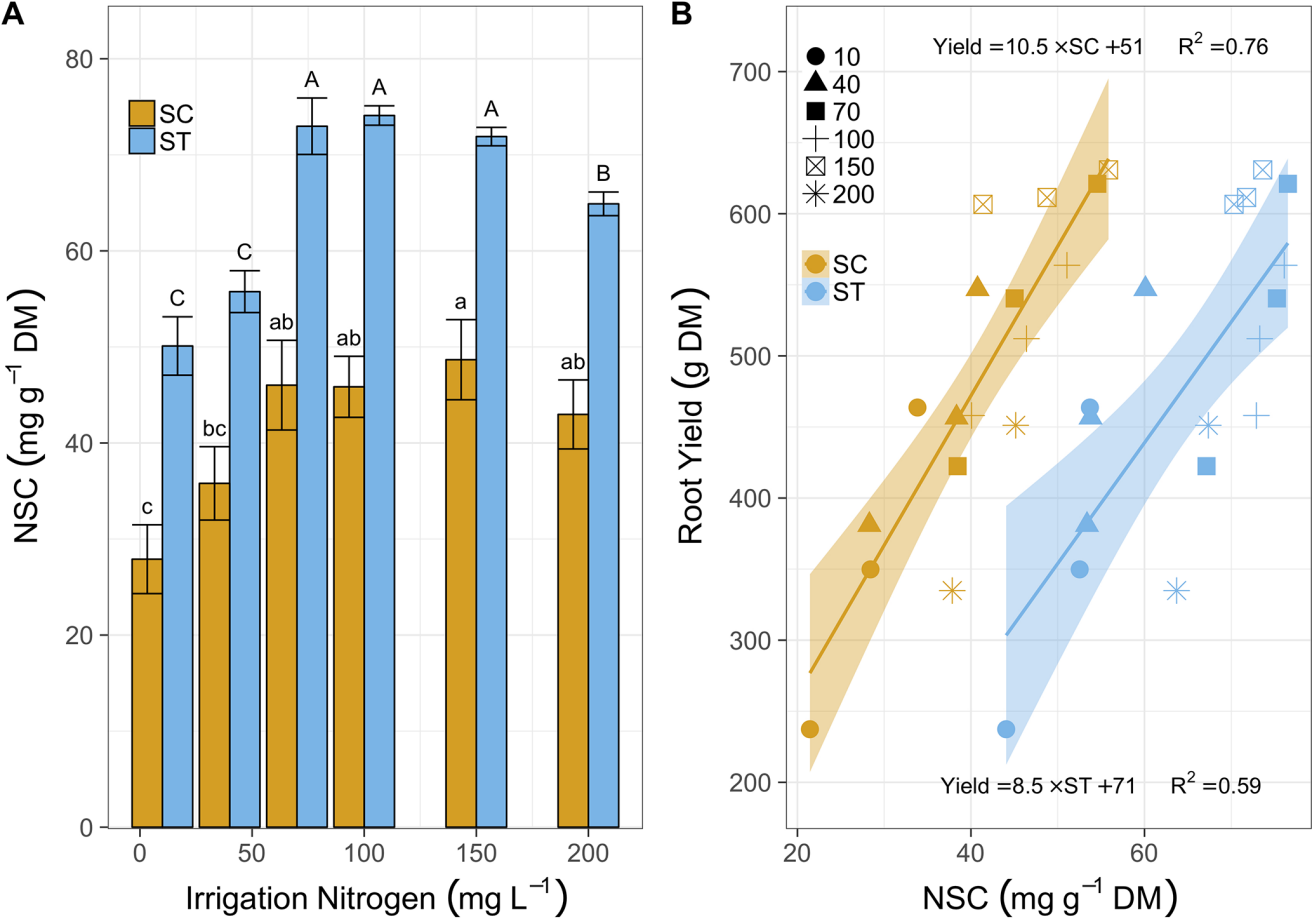
**(A)** Soluble carbohydrates (SC, mg g^-1^ DM, yellow bars) and starch (ST, blue bars) in leaves sampled after 81 days of treatment with irrigation N from 10 to 200 mg L^-1^. Bars represent means (3 biological replicates), error-bars denote SE, and letters stand for statistical differences (One-way ANOVA and Tukey-LSD, lowercase for SC and upper for starch, P < 0.05). **(B)** The relations between cassava root yield (g DM) and SC (yellow shapes) or starch (blue shapes) after 120 days of N fertigation ranging between 10 and 200 mg L^-1^ (various shapes). Lines (yellow for SC and blue for starch) illustrate positive linear regressions (equations and R^2^ are listed above line for SC and below the line for starch) and the ribbons mark the 95% confidence intervals.

Cassava leaves induced SC levels from 28 to 46 mg g^-1^ DM as irrigation N increased from 10 to 70 mg L^-1^ (**Figure 3A**). At 150 mg N L^-1^, SC levels increased again, slightly, to 48 mg g^-1^, but dropped to 43 at 200 mg N L^-1^. Starch followed the same trend in a bigger scale as it increased from 51 mg g^-1^ (10 mg L^-1^) to ∼72 mg g^-1^ (for 70, 100, and 150 mg N L^-1^ treated plants) and then reduced to 65 mg g^-1^ at 200 mg N L^-1^ irrigation. Strikingly, this resembled the trend of root mass accumulation. In fact, both SC and starch levels were linearly correlated to cassava root production, independent of N irrigation levels (**Figure 3B**). Yields averaged 311 g plant^-1^ in plants irrigated with 10 mg N L^-1^, with 28 g mg^-1^ SC and 50 mg g^-1^ starch, and doubled to 616 g plant^-1^ for plants irrigated with 150 mg N L^-1^, which increased SC and starch to 50 and 72 mg g^-1^, respectively. Yet once more, the 200 mg N L^-1^ treatment had negative effects on cassava plants as their leaves reduced SC to 40 mg g^-1^, starch was down to 65 mg g^-1^, and their yield dropped to an average of 393 g plant^-1^ (a 36% yield loss).

## Discussion

With the increasing importance of cassava production and the prominent role fertigation plays in modern farming, we studied cassava’s physiological responses to different irrigation N levels. Our research supports the notion that an excessive use of N would favor shoot vegetative growth and compromise yields. We were determined to assess whether cassava’s nutritional status could be evaluated by the traditional N levels in diagnostic leaves and concluded that we need an alternative indicator of productivity for root crops.

**Nitrogen application increases cassava yields, but it might also promote shoot development over root growth and make N application ineffective**. Nitrogen is arguably the biggest promotor of yields in modern farming (Spiertz, 2010), and it has shown the potential to increase cassava yields by 100%. Yet, in low N availability (below 70 mg L^-1^), it led to contrasting effects. Additional N in such conditions, while probably stimulating canopy growth, photosynthesis, and NSC accumulation (Leuning et al., 1995; Millard and Grelet, 2010), did not translate to cassava root growth (and more yields for that matter). Apparently, carbon transport is limited in N-deficient cassava plants (Cruz et al., 2003a), which implies that root-crops might require substantial N application before fertilization pays off in increased yields. Higher N levels, between 70 and 150 mg N L^-1^, were effective and increased cassava yields. At these N levels, plants benefit from acquiring more minerals, and therefore supply ample carbohydrates and recycled N to root growth (Thornley, 1972; Millard and Grelet, 2010), which is yield in cassava. Yet higher N concentrations, i.e., 200 mg L^-1^, underlined a most striking concern - excessive N can lower yields. In our research, the high N could lead to high levels of ammonium (due to nitrogen application in inert media with a fixed ammonium-nitrate ratio) and possible toxicity (Marschner and Marschner, 2012). Alternatively, high N levels could, somewhat counterintuitively, limit nitrate availability to roots, as it is probably assimilated in the shoot where nitrate reductase is most active (Cruz et al., 2004). Yet nearly similar limits to N fertigation in soil (70 to 130 mg L^-1^) were previously reported for an important tuber crop – potato (Papadoppoulos, 1988), with similar concerns at higher N levels, suggesting this is a broader phenomenon. Another explanation to why cassava yielded less, is that high N conditions could represent favorable growing conditions, trigger competition for light or space, and drive plants to promote vegetative development over root growth (Davis et al., 1999; Cruz et al., 2003a). In such a case, further fertigation is ineffective, monetarily wasteful, and environmentally concerning. Finally, cyanide is still a concern in some cassava cultivars (albeit not in the variety we studied) and it should be assessed in future studies because it is often attributed to high nitrogen availability (Burns et al., 2012). Yet none of these conditions could be recognized through the vitality of cassava growth, and therefore precise cassava N application calls for physiological indicators.

Traditional leaf mineral analyses fail to indicate whether cassava fertigation will promote yields (i.e., root growth) because total N in leaves hardly responds to changes in irrigation N. Generally, N concentration in leaves is not an optimal reference because it changes between species and throughout the growing season (Reich et al., 1991). Additionally, N uptake by mature leaves is negligible (Marschner and Marschner, 2012), and new N is primarily allocated through the transpiration stream to the younger leaves. In cassava, the growth rate is rather steady throughout the growing season (Keating et al., 1982), environmental water deficits and transpiration rate govern N deposition in the young, fully developed leaves (i.e., diagnostic leaves). Naturally, bigger plants and higher transpiration rates promote N accumulation in leaves. Yet high N availability will not necessarily promote soluble N accumulation in cassava leaves (Cruz et al., 2004), possibly because stunted plants (even due to N deficiency) grow less canopy and therefore accumulate N in their fewer, younger, leaves. Therefore, without extensive climatic, phenological, and variety-specific experiments, leaf mineral analyses are but arbitrary numbers and not a reference to precise farming. Nitrate in the petioles on the other hand, which represents the transient xylem sap solute composition (Wu et al., 2007), did denote the differences in the irrigation N. Nevertheless, sap nitrate is also affected by the strength of N sinks (mainly new growth), and therefore it increases as plants grow, transpire more, and transport more residual N towards the premature leaves. Overall, it is difficult to determine N availability by its deposition without extensive empirical studies, but it is obvious that N uptake is tightly linked to transpiration and may affect its byproducts.

Nitrogen application affects cassava plants’ assimilation and allocation of photosynthates. Low N availability biochemically limits carboxylation and photosynthesis of cassava plants (Cruz et al., 2003b). Accordingly, as irrigation N increased from 10 to 70 mg L^-1^, cassava plants supported more photosynthesis, which translated into increasing SC levels in the canopy. Yet starch did not increase (53 ± 4 mg g^-1^), and that implies that these plants have not reached their photosynthetic capacity and that the canopy could not spare resources. Only when leaves reached a sugar threshold value of 47 ± 4 mg g^-1^ did they begin to increase starch. They actually sustained peak starch levels of 73 ± 2 mg g^-1^ for 70, 100, and 150 mg N L^-1^, which outlined the N treatments where fertigation was effective. As cassava is a starchy root crop, N at these levels promoted the allocation of these reserves to the roots (possibly by elevating ADP-glucose pyrophosphorylase activity (Ihemere et al., 2006) and improved yields. However, at 200 mg N L^-1^, a reduction in petiole nitrate demonstrated that these cassava plants could not fully support their canopy and needed to inhibit transpiration. Consequently, leaf stomatal conductance [supposedly not a limiting factor at low N conditions (Cruz et al., 2003b)], which linearly increased with irrigation levels so far, restricted water loss at 200 mg N L^-1^ irrigation. Most importantly, this small change in conductance led to a dramatic drop in photosynthesis (25%) and altered the carbohydrate availability to both roots and canopy. In fact, reduced root growth at fertigation of 200 mg N L^-1^ could have suspended carbohydrates in the canopy and acted as feedback inhibitors to photosynthesis (Goldschmidt and Huber, 1992). Moreover, former reports suggest that SC could increase dramatically (despite no photosynthetic limitations) at only 90 mg N L^-1^ (Cruz et al., 2003a). These shifts in carbohydrate status, easily, non-destructively, and inexpensively detectable at the leaf level, can actually disclose the cassava’s nutritional status and indicate whether the fertigation is supporting yield. Moreover, these are physiological traits at the whole plant level that could be further studied in the field to produce an applied protocol and improve N application in cassava crops.

## Conclusions

**Figure 4:**
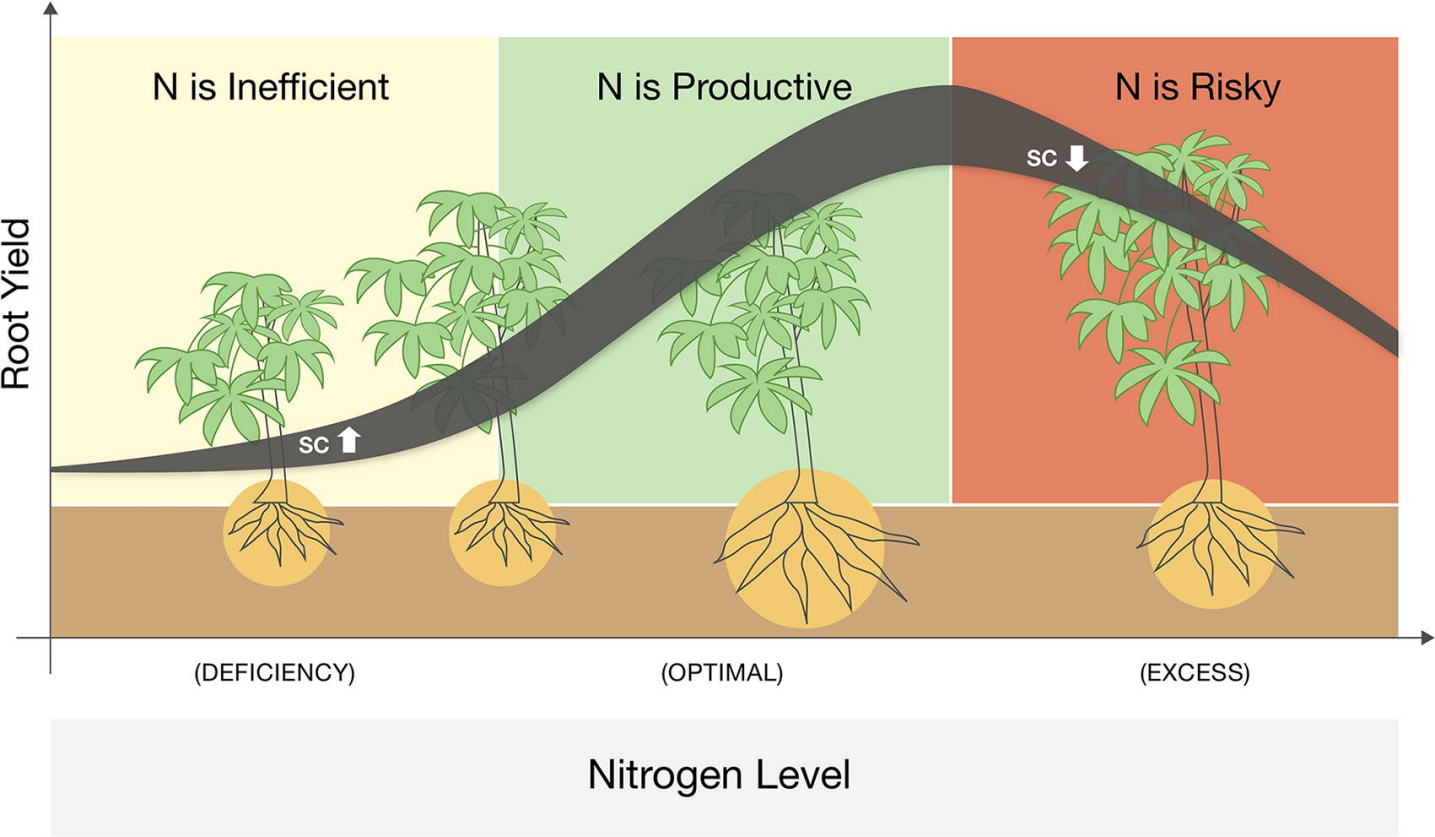
A schematic summary of a three-phase response of cassava to nitrogen fertigation ranging from deficient to excessive. Line denotes the yield response while its width represent changes in sugar (soluble carbohydrates, SC) levels in the leaves. Plant sizes illustrate the changes in roots vs. shoots biomass and the background colors mark were N application is infective (yellow), productive (green), or wasteful and risky (red).

We identified three phases of cassava response to N fertigation that could be adjusted and extrapolated to other annual root crops (**Figure 4**). At low N levels, plant growth is strictly inhibited, and N addition dramatically enhances their photosynthesis, sugar levels, and aboveground vegetative growth. Yet the main energy investment at this phase is in the canopy, and root development (not yet a limiting factor) is not promoted. For cassava (or any root-crop), N application in this range is potentially wasteful. At medium N levels, plants reach a threshold of vegetative growth and cellular sugar capacity and translocate resources (both C and N) to the roots. Such roots develop faster and reciprocate with more water and nutrients to the demanding canopy. These are the conditions where N applications are highly effective. Yet at higher N levels the plant canopy becomes too demanding, and due to additional environmental constraints (e.g., irrigation regime, soil water capacity, or peak midday temperatures), roots fail to support it. In such conditions, plants become conservative, minimize water loss, photosynthesis, and carbohydrate redistribution. In this phase, farming inputs (both water and nutrients) exceed the plant’s productive capacity, and further fertigation becomes an irresponsible practice that exhausts resources and risks the environment.

## Data Availability

The datasets generated for this study are available on request to the corresponding author.

## Author Contributions

UY, NL, and SR conceptualized the research and guided the work, documentation, and analysis of the results. JOO set the experiments, conducted the field measurements, and documented the results. OS and UY guided the lab analysis and drafted the manuscript, while JOO, NL, SR, UY, and OS iteratively revised the manuscript until it was ready for submission.

## Conflict of Interest Statement

The authors declare that the research was conducted in the absence of any commercial or financial relationships that could be construed as a potential conflict of interest.
